# Supplementary material for: Content-rich biological network constructed by mining PubMed abstracts
Source: BMC Bioinformatics. 2004 Oct 8;5:147. doi: 10.1186/1471-2105-5-147 (PMC528731; doi:10.1186/1471-2105-5-147)
Supplement: Additional File 2 — The original results of the above study (non-essential files are deleted to keep the file size under the limit set by BMC bioinformatics). [file 1471-2105-5-147-S2.bz2 › chilibotAdditionalFile2/dip05/348ID7721786E4496/html/IL4R_IL13.html]

 


 **IL4R** and **IL13** 
  
Found 306 abstracts in PubMed, retrieved 05.  
 

 What does Google say? 
 PDF only 
| .edu only 

---

**Interactive relationship** (e.g. stimulation, inhibition, etc)

**Non-interactive relationship** (e.g. studied together, co-existance, homology, etc.)

- Using an in vitro cell culture system, we investigated how the key regulatory cytokines interleukin 4  [ **IL4R** ]  IL 4, IL 10, IL 13  [ **IL13** ] , and TGF beta might modulate the effect of IFN gamma in inducing resistance to infection in enterocyte cell lines.  Ref: 12874337 Infect Immun, 2003
- The roles of interleukin 4  [ **IL4R** ]  IL 4 and IL 13  [ **IL13** ]  in the regulation of immunity to Leishmania donovani infection are still poorly understood.  Ref: 12874364 Infect ImmunInfect Immun, 2003
- Neutralizing antibodies against human RANTES, MIP 1alpha, MIP 1beta, alpha interferon IFN alpha, IFN beta, IFN gamma, interleukin 4  [ **IL4R** ]  IL 4, IL 10, IL 13  [ **IL13** ] , IL 16, MCP 1, MCP 3, tumor necrosis factor alpha TNF alpha, or TNF beta failed to reverse the HIV 1 suppressive activity.  Ref: 12885891 J VirolJ Virol, 2003
- Here we show that mice lacking interleukin 12p35 IL 12p35 following T. crassiceps infection, failed to mount a Th1 response, but developed a strong Th2 type response, produced higher levels of IgG1, IgE, interleukin 4  [ **IL4R** ] , interleukin 5 as well as interleukin 13  [ **IL13** ]  than wild type mice, and became highly susceptible to the larval stage of this cestode.  Ref: 12814650 Int J Parasitol< MedlineTA>Int J Parasitol, 2003
- Interleukin 4  [ **IL4R** ]  and IL 13  [ **IL13** ]  activated phosphorylation of mitogen activated protein kinase in LS174T cells.  Ref: 12848848 Immunol Cell Biol, 2003
- In contrast, similarly infected CD40 deficient BALB c mice CD40 displayed impairment of both Th1 and Th2 type responses associated with low levels of interferon gamma as well as IgE, interleukin 4  [ **IL4R** ] , interleukin 5 and interleukin 13  [ **IL13** ] , but efficiently controlled T. crassiceps infection.  Ref: 12814650 Int J Parasitol< MedlineTA>Int J Parasitol, 2003
